# Kohlschütter-Tönz Syndrome – Report of an additional case

**DOI:** 10.4317/jced.51018

**Published:** 2013-04-01

**Authors:** Wilfredo A. González-Arriagada, Román Carlos-Bregni, Elisa Contreras, Oslei P. Almeida, Marcio A. Lopes

**Affiliations:** 1 DDS, MSc. Oral Diagnosis Department, Semiology and Oral Pathology, Piracicaba Dental School, State University of Campinas (UNICAMP), Piracicaba, Sao Paulo, Brazil; 2 Assistant professor. Oral Pathology and Diagnosis, Facultad de Odontología, Universidad de Valparaíso, Valparaíso, Chile; 3 DDS. Oral Pathology Section, Centro Clínico de Cabeza y Cuello/Hospital Herrera Llerandi, Guatemala City, Guatemala; 4 DDS, PhD. Oral Diagnosis Department, Semiology and Oral Pathology, Piracicaba Dental School, State University of Campinas (UNICAMP), Piracicaba, Sao Paulo, Brazil

## Abstract

Kohlschütter-Tönz Syndrome is a rare disorder clinically characterized by amelogenesis imperfecta, epilepsy and progressive mental deterioration. We present an additional case of this syndrome of a nine year-old boy who was referred by pigmented teeth. The mental deterioration was associated with speech delay, impulsive behavior, attention-deficit/hyperactivity disorder, and learning problems. The physical examination revealed a reduction of lower third, slightly palpebral fissures, low ear and hair implantation, coarse hair and hypertrichosis. The intraoral examination showed alteration in teeth pigmentation diagnosed as amelogenesis imperfecta. Although rare, the present case report illustrates a syndrome that has dental anomalies and systemic alterations. It is important to recognize this syndrome as early as possible and paediatric dentist may contribute to the diagnosis and consequently to better manage the patients.

** Key words:**Kohlschütter-Tönz syndrome, amelogenesis imperfecta, seizures, mental deterioration.

## Introduction

Kohlschütter-Tonz Syndrome is an uncommon disorder that has been associated with an autosomal recessive inheritance. Clinically it is characterized by amelogenesis imperfecta, epilepsy and progressive mental deterioration. Other clinical manifestations such as myopia, ventricular enlargement, dry skin and altered thumbs/toes also have been described (1-5).

This disorder was described by Kohlschütter et al. (1) in 1974 and forty-three cases have been reported in the English-language literature (1-11). In the present paper we describe an additional case with the typical features of this syndrome and a brief literature review.

## Case Report

A nine-year-old boy was referred for evaluation because of pigmentation of his teeth. Her mother stated that her pregnancy was of high risk, and that she took fenobarbital during this period. The boy was born with a normal birth weight of 4.500 g (10 lbs). His past medical history revealed that he underwent amygdalectomy when he was 4 years-old. When he was 8 years-old he presented generalized tonic-clonic seizure, and was treated with valproic acid. A brain angiotomography was performed and any alterations were found. However, the boy manifested mental deterioration and hyperactivity. The patient is in neurological follow-up because speech delay, impulsive behavior, attention-deficit/hyperactivity disorder, and learning problems. He was treated with atomoxetine hydrochloride in association with risperidone, presenting improvement in learning and behavior. The mother also reported that he has excessive sweating.

**Figure 1:**
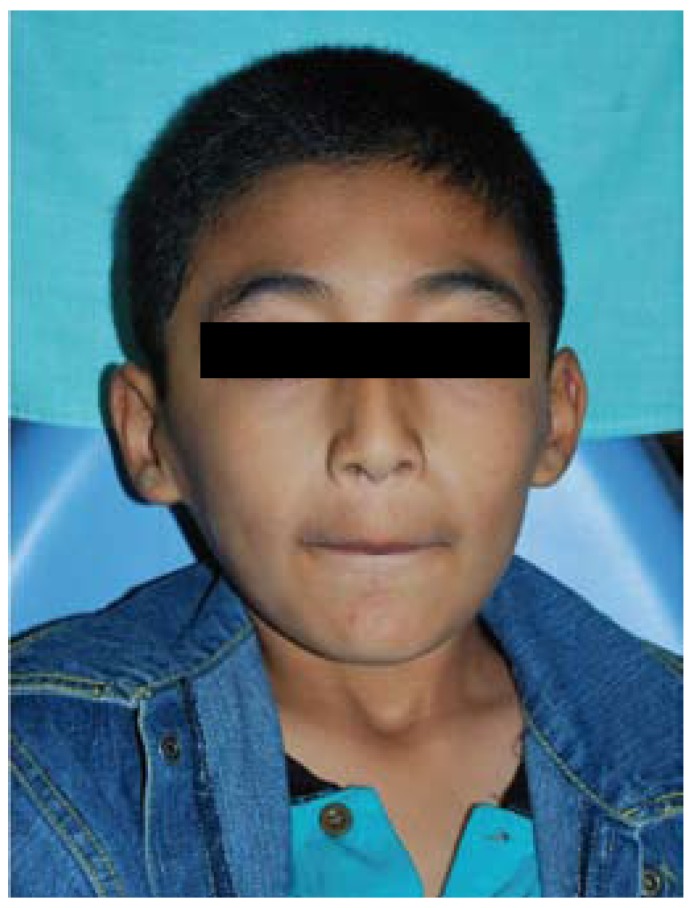
Extraoral examination showing low ear and hair implantation, and a slight reduction in the lower third of the face.

**Figure 2:**
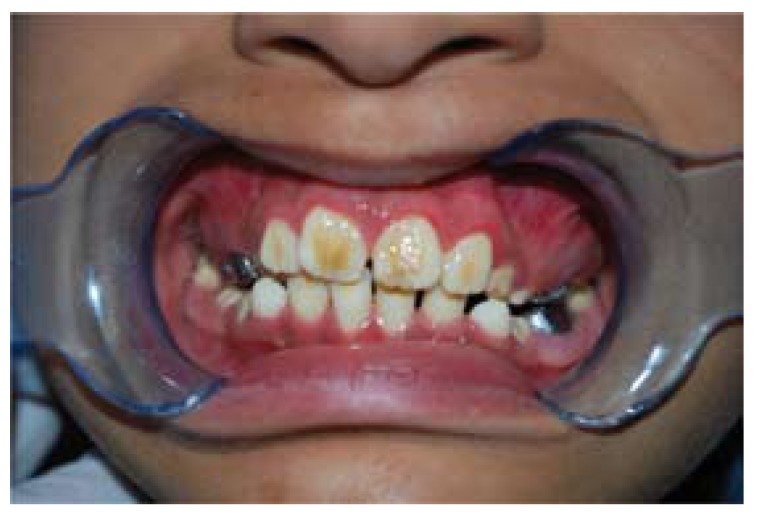
Intraoral examination showing metal molar crowns and tooth discoloration.

In the extraoral examination (Fig. 1) the patient showed a symmetric face but with a reduction of lower third, slightly palpebral fissures, low ear and hair implantation, coarse hair and hypertrichosis. The intraoral examination showed crowding teeth with generalized enamel defects in all the teeth, suggesting the clinical diagnosis of amelogenesis imperfecta with a yellow-brownish coloration, and high incidence of caries (Fig. 2). The orthopantomography showed no alterations and normal teeth eruption. Blood and urine test had normal values. Electrolyte analysis of chloride presented a slight elevated value (107.2 mEq/L; ref. 95-106 mEq/L). The ophtalmological evaluation did not reveal any alteration.

## Discussion

Previously, forty-three cases of Kohlschütter-Tönz Syndrome have been reported in the English-language literature ( Table 1). The typical characteristics of this syndrome (amelogenesis imperfecta, early onset seizures and progressive mental retardation) have been observed in all the reported cases, but with a variable expressivity (1-5,7-11). The dental abnormalities affect both primary and secondary dentition (10). The enamel malformation is associated with a high susceptibility to caries (9), and this can be observed in intraoral pictures of the current patient. Delayed eruption have been reported in some patients (9). This disorder is always diagnosed in young child (0-4 years-old) when they present the first convulsion (1). These seizures are usually treatment resistant to various anti-epileptic agents, and the patient present psychomotor delay or regression in infancy, associated to spasticity of the lower and upper limbs. Severe progressive psychomotor decline and fatal outcome has been reported in some patients (9). The inherited cause of this disorder is supported by the reported familial cases and consanguinity, suggesting autossomal recessive inheritance, confirmed by recent reported findings (1,3-5,9). In our case the parents have not consanguinity and the patient does not have brothers. The cause of the progressive mental deterioration is unknown, but have been associated with the seizures, that could cause a brain damage difficult to be controlled (2). Musumeci et al. (4), suggested that this syndrome is a neurodegenerative disease caused by a metabolic disturbance, but any metabolic alteration was reported. Laboratoy blood and cerebrospinal fluid, and histopathological reported findings are unremarkable or inconclusive (1,6,9). Our patient presents the characteristics of the Kohlschütter-Tönz syndrome in a low grade, being the mental deterioration the most severe problem. The mother of the patient affirmed that there were not relatives affected by amelogenesis imperfecta or another sign of the disease, being considered as an isolated case without familiar inheritance.

**Table 1:**
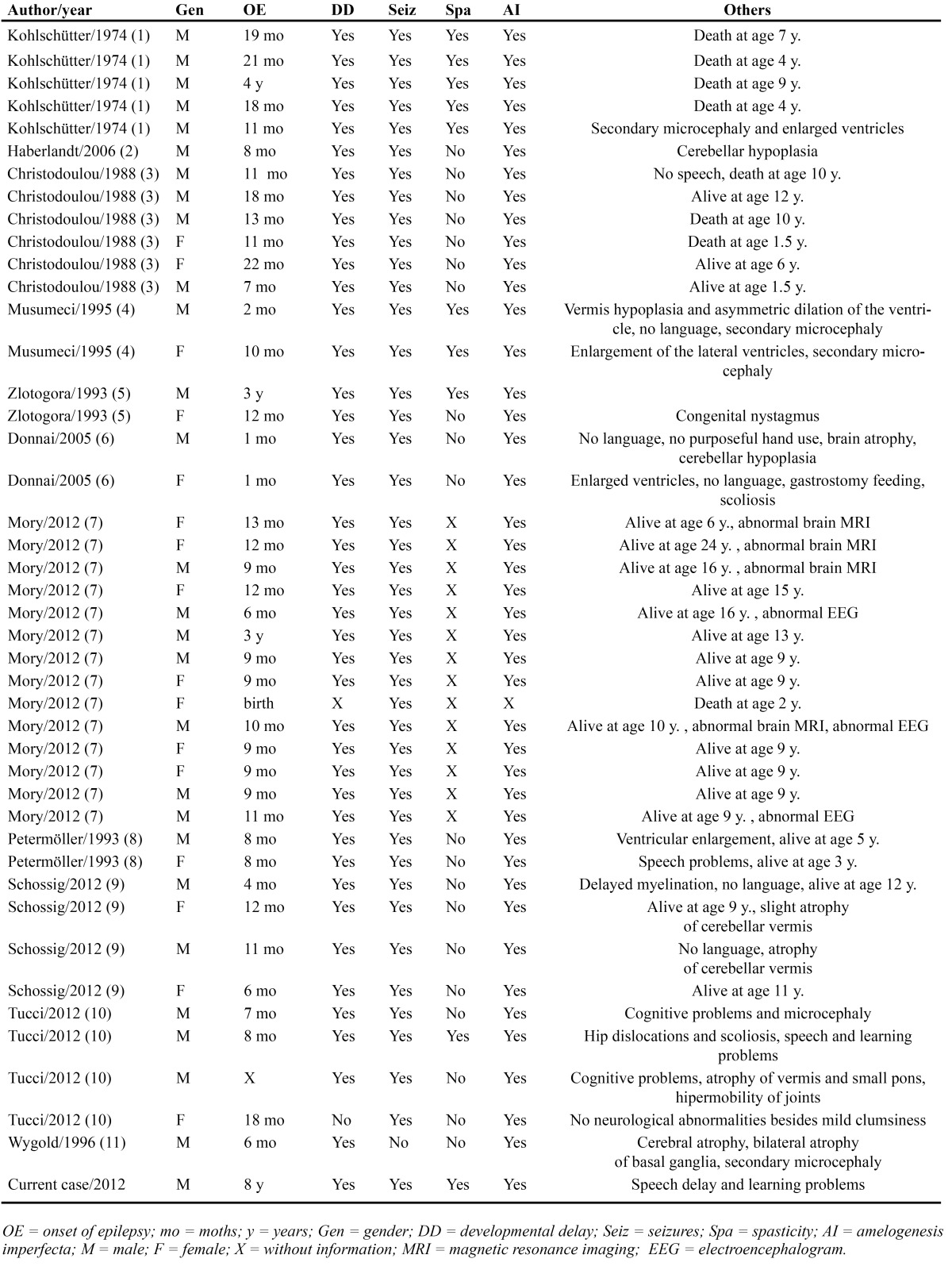
Previously reported cases of Kohlschütter-Tönz Syndrome, including clinical features.

Associated anomalies are described but they are not constant (9). Reported patients have minor physical abnormalities such as small stature, microcephaly, scoliosis, broad thumbs and toes, café-aut-lait spots and vitiligo, bristly hair, deeply set eyes, palpebral fissures, small ears, short nose, concave nasal ridge and smooth philtrum (1,4,6,9-11). Magnetic resonance imaging (MRI) and computed tomography (CT) findings were reported in previous cases, showing additional abnormalities in brain (9). These image scans were not performed in our patient. Recently, mutations on RODGI in chromosome 16 (MIM# 614574), a gene that encodes a protein of unknown function, and genetic heterogeneity was reported (10,12). The gene has orthologs in many species, including Drosophila melanogaster and it shows high expression levels in various human brain regions (7,12). A Drosophila mutant of this gene showed a possible deficiency in olfactory memory (7). The differential diagnosis can include Rud syndrome, tuberous sclerosis, mucopolysaccharidosis, oculodentodigital dysplasia and isolated or syndromic amelogenesis imperfecta, but it is very limited and these disorders can be easily distinguished because mainly the progression (2,4). The treatment of these patients is based in the following by the neurologist for the epilepsy and the mental retardation and the dentist because the high risk of caries in the patients with amelogenesis imperfecta. Because the rarity of this disorder the report of new cases is necessary for a better clinical and genetic characterization.
